# LncRNA-Gm9866 promotes liver fibrosis by activating TGFβ/Smad signaling via targeting Fam98b

**DOI:** 10.1186/s12967-023-04642-1

**Published:** 2023-11-02

**Authors:** Xiaomin Liao, Xianxian Ruan, Peishan Yao, Dan Yang, Xianbin Wu, Xia Zhou, Jie Jing, Dafu Wei, Yaodan Liang, Taicheng Zhang, Shanyu Qin, Haixing Jiang

**Affiliations:** 1 Department of Gastroenterology, The First Affiliated Hospital of Guangxi Medical University, No. 6, Shuangyong Road, Nanning, 530021 Guangxi China; 2 Department of Gastroenterology, The Affiliated Hospital of Southwest Medical University, Luzhou, 646000 Sichuan China; 3 Department of Gastroenterology, The Wuming Affiliated Hospital of Guangxi Medical University, Nanning, 530000 Guangxi China; 4 Department of Emergency, People’s Hospital of Guizhou Province, Guiyang, 550000 Guizhou China

**Keywords:** lncRNA, Gm9866, Liver fibrosis, Apoptosis, Fam98b

## Abstract

**Objective:**

The exact mechanism and target molecules of liver fibrosis have remained largely elusive. Here, we investigated the role of long noncoding RNA Gm9866(lncRNA-Gm9866) on liver fibrosis.

**Methods:**

The transcription of lncRNA-Gm9866 in activated cells and mouse fibrotic livers was determined by quantitative polymerase chain reaction (qRT-PCR). The effects of lentivirus-mediated knockdown or overexpression of lncRNA-Gm9866 in liver fibrosis were examined in vitro and in vivo. Furthermore, bioinformatics analysis, cell samples validation, fluorescence in situ hybridization (FISH) co-localization, RNA binding protein immunoprecipitation (RIP), actinomycin D test and Western blot (WB) were carried out to explore the potential mechanism of lncRNA-Gm9866.

**Results:**

The expression of α-smooth muscle actin (α-SMA), Collagen I (COL-1) and lncRNA-Gm9866 were significantly increased in tissues and cells. Overexpressing lncRNA-Gm9866 promoted the activation of hepatic stellate cells (HSCs). Silencing lncRNA-Gm9866 inhibited the activation of HSCs and transforming growth factor-β1 (TGFβ1) induced fibrosis. Overexpressing lncRNA-Gm9866 promoted hepatocytes (HCs) apoptosis and the expression of pro-fibrogenic genes, inhibited the proliferation and migration of HCs. Knockdown of lncRNA-Gm9866 inhibited the apoptosis of HCs, the expression of pro-fibrogenic genes, TGFβ1 induced fibrosis and the occurrence of carbon tetrachloride (CCl4)-induced liver fibrosis, and promoted the proliferation and migration of HCs. Mechanistically, lncRNA-Gm9866 may directly bine with Fam98b. Silencing Fam98b in stably overexpressing lncRNA-Gm9866 cell lines reversed the increase of pro-fibrogenic genes and pro-apoptotic genes, fibrosis related pathway protein TGFβ1, Smad2/3, p-Smad2/3 and Notch3 induced by overexpressing lncRNA-Gm9866.

**Conclusions:**

LncRNA-Gm9866 may regulate TGFβ/Smad and Notch pathways by targeting Fam98b to regulate liver fibrosis. LncRNA-Gm9866 may be a new target for diagnosis and treatment of liver fibrosis.

## Background

Liver fibrosis and cirrhosis are the results of most chronic liver injuries, and are a common and intractable global clinical challenge. Therefore, it is imperative to develop anti-fibrosis strategies suitable for liver fibrosis. The development of liver fibrosis requires significant changes in the quantity and quality of the extracellular matrix (ECM) in the liver. Activated hepatic stellate cells (HSCs) are the main producers of new matrix in fibrosis [1, 2]. In the fibrotic liver, static HSCs transdifferentiate into proliferating, migrating, and contracting myofibroblasts, showing fibrogenic transcription and secretion characteristics (so-called "cell activation"). Hepatic stellate cells are the main driver of liver fibrosis and the key target of liver fibrosis [3]. Exposure of activated HSCs to relaxin results in a concentration-dependent reduction in collagen synthesis and deposition and alleviates liver fibrosis [4]. Liu et al. found that praziquantel inhibited the activation of HSCs and expression of the collagen matrix by increasing the expression of Smad7, and significantly reduced carbon tetrachloride (CCl_4_)-induced liver fibrosis in vivo and in vitro [5]. Recent reports have demonstrated that inducing HSCs senescence and promoting HSCs apoptosis inhibits liver fibrosis [6, 7]. Although HSCs play a key role in liver fibrosis, hepatocytes (HCs) are the dominant in the liver, and impaired apoptosis and proliferation of HCs are also generally considered to trigger fibrosis by activating HSCs in persistent liver injury. Therefore, it is believed that one solution to liver fibrosis is to inactivate HSCs and inhibit HC apoptosis [8, 9]. A previous study indicated that camellia oil inhibits the activation of HSCs by reducing HCs apoptosis, thereby reduced CCl4 induced liver fibrosis in mice [10]. Another study reported that insulin growth factor binding protein (IGFBPrP1) induced liver fibrosis by mediating HSCs activation and HCs apoptosis using in vivo and in vitro experiments [11]. The latest research shows that in male mice, hepatocyte peroxisome proliferators-activate receptors γ (PPAR γ) negatively regulate methionine metabolism and promote the progress of fibrosis[12, 13].

Long noncoding RNAs (lncRNAs), a form of noncoding RNA greater than 200 nucleotides in length, can directly participate in epigenetic, transcriptional, and post-transcriptional regulation of mRNAs; lncRNAs also regulate target genes by competitive inhibition of miRNAs by sponge action [14–16]. Several lncRNAs have been linked to the pathogenesis of liver fibrosis [8, 17]. For example, Zhang et al. detected the expression of lncRNAs in a mouse model of liver fibrosis by sequencing technology and identified a new lncRNA, lncRNA-LFAR1, that could activate the TGFβ and Notch pathways to promote HSCs activation and development of liver fibrosis [8]. Another study revealed that overexpression of lncRNA-H19 downregulated the expression of ZEB1 (E-box-binding homeobox 1) and upregulated expression of EpCAM (epithelial cell adhesion molecule) and SRY (sex determining region Y)-box 9 in a mouse model of cholestatic liver fibrosis [18]. LncRNA-SNHG7 regulates activity of the miR-29b/DNMT3A axis, affecting the activation, autophagy, and proliferation of HSCs in liver fibrosis [19]. Our previous study demonstrated that macrophage polarization is related to liver fibrosis [20], and lncRNA-Gm9866 has been closely linked to macrophage polarization through bioinformatics analysis [21] and our recent research [22]. Therefore, we speculate that lncRNA-Gm9866 is related to liver fibrosis. Although the field of long noncoding RNAs is developing rapidly, investigation of lncRNA-Gm9866 and liver fibrosis is lacking.

In this study, we detected the expression of lncRNA-Gm9866 in the liver of fibrotic mice and normal mice by quantitative polymerase chain reaction (qPCR). LncRNA-Gm9866 was specifically upregulated in liver fibrosis. This upregulation was caused by TGFβ, and promoted HSCs activation and TGFβ-induced HCs apoptosis. Mechanistically, we show that lncRNA-Gm9866 can directly bind Fam98b, stabilizing Fam98b mRNA; silencing Fam98b rescued the pro-fibrogenic and pro-apoptosis effects of lncRNA-Gm9866 overexpression. Western blot showed that lncRNA-Gm9866 can promote Smad2/3 phosphorylation and regulate the Notch pathway. Overall, our data suggest that knockdown of lncRNA-Gm9866 can significantly inhibit HSCs activation and HCs apoptosis, thereby inhibiting CCl4-induced liver fibrosis in mice. This study provides a possible mechanism and therapeutic approach for the diagnosis and treatment of liver fibrosis.

## Materials and methods

### Animals and experimental design

About 6-week-old C57BL/6 male mice (initially weighing 18–20 g) were purchased from the Laboratory Animal Center (Guangxi Medical University). All animals were housed under a controlled environment (12 h light/dark cycle; temperature: 22−24 ℃) and received water ad libitum. Mice were divided into three groups, with six mice in each group: (1) group 1 were control animals that received a vehicle (olive oil); (2) the mice in group 2 were injected intraperitoneally with CCl4 (Sigma-Aldrich, St. Louis, MO, USA) for 4 weeks to induce liver fibrosis; and (3) mice in group 3 were injected intraperitoneally with CCl4 for 6 weeks to induce liver fibrosis. For the CCl4 induced mouse liver fibrosis model, 24 C57BL/6 mice were randomly divided into four groups: (A) olive oil + lenti-NC (NC for negative control, n = 6), (B) CCl4 + lenti-NC (NC + CCl4, n = 6), (C) olive oil + lenti-shGm9866 (shGm9866, n = 6), and (D) CCl4 + lenti-shGm9866 (shGm9866 + CCl4, n = 6). One week after the first injection of CCl4 (4 ml/kg), NC or shGm9866 lentivirus (1*10^7^ TU/mL, 300 µL each mouse) was injected through the tail vein. The NC + CCl4 and shGm9866 + CCl4 groups were administered 20% CCl4 dissolved in olive oil twice a week (4 ml/kg) for another 4 weeks after the lentivirus was injected. Animals in the NC and shGm9866 groups were injected with the same amount of olive oil. After 4 weeks of treatment with CCl4, all mice were euthanized with chloral hydrate anesthesia.

### Histological and immunochemical analyses

Tissue sections were prepared at a thickness of 4 μm and stained with hematoxylin, eosin, and Masson according to standard procedures and liver histology was assessed using an Olympus BX53 + DP80 upright fluorescence microscope imaging system (Olympus, Tokyo, Japan). According to the microscopy, three sections were chosen from each group for IHC analysis. Briefly, the sections were dewaxed, washed with water, placed in citric acid antigen repair buffer (pH 6.0) for antigen repair, and washed with phosphate-buffered saline (PBS). The sections were placed in 3% hydrogen peroxide solution, incubated at room temperature in the dark for 25 min, and washed with PBS. Then, 3% bovine serum albumin (BSA) was added to block at room temperature for 30 min. The slides were treated with primary antibodies against α-SMA (1:2000, Servicebio, Wuhan, China, GB111364) and COL-1 (1:1000, Servicebio, Wuhan, China, GB11022) overnight at 4 °C and then incubated with the appropriate secondary antibody (1:200, Servicebio, Wuhan, China, GB23303) (HRP-labeled goat anti-rabbit). Reaction products were visualized using diaminobenzidine (DAB, Servicebio, Wuhan, China) and monitored by microscopy.

### Cell culture

AML12 mouse hepatocytes, which are immortalized normal mouse hepatocytes, were purchased from WHELAB (Shanghai, China). The cells were cultured in Dulbecco’s Modified Eagle’s Medium (Gibco, Gaithersburg, MD, USA) supplemented with 1% ITS Liquid Media Supplement, 40 ng/mL dexamethasone, 10% heat-inactivated fetal bovine serum (BI, Israel; VivaCell, Shanghai, China), 1% penicillin/streptomycin, and bicarbonate at 37 °C under 5% CO_2_. AML12 cells were divided into five groups: control, transforming growth factor beta1 (TGFβ1, Novoprotein, Suzhou, China) at concentrations of 5, 10, and 20 ng/mL. Each treatment was for an additional 48 h. JS-1 mouse HSCs were purchased from Otwo Biotech (Shenzhen, China) and cultured in Dulbecco’s modified Eagle’s medium (Gibco, Gaithersburg, MD, USA), 10% heat-inactivated fetal bovine serum (BI, Israel; VivaCell, Shanghai, China), 1% penicillin/streptomycin, and bicarbonate at 37 °C under 5% CO_2_.

### CCK-8 and EdU assay

Proliferation was monitored via CCK-8 assays (Meilun Biotechnology, Dalian, China). In brief, AML12 cells were inoculated into 96-well plates and cultured at 37 ℃ for 24 h. Then, 10 µL of CCK-8 enhanced solution was added to each well and incubated for 1 h at 37 ℃. The absorbance at 450 nm was then determined with a microplate reader and each group had three vice-holes. Proliferation was monitored via EdU assays (Ribobio, Guangzhou, China). In brief, cultured AML12 cells were incubated with 50 uM EdU solution for 24 h, followed by fixation by 4% paraformaldehyde for 30 min and permeation using 0.5% TritonX-100 for 10 min. Afterwards, the treated cells were stained by Apollo fluorescent dyeing solution and Hoechst 33,342 for 30 min and observed with an inverted fluorescence microscope (Nikon, Japan).

### Apoptosis assay

TUNEL staining was employed to test apoptosis of AML12 cells (Meilun Biotechnology, Dalian, China). The experimental methods rigorously followed the manufacturer’s instructions. Fluorescence microscope was used to observe the features of apoptosis. AML12 Cells stained by TUNEL were considered apoptotic. The total number of apoptotic cells was calculated using DAPI staining.

### Cell migration assay

Migration of AML12 cells was evaluated using the Transwell assay (Corning, NY, USA, 3396). Briefly, 10 × 10^4^ cells were seeded in the upper chamber. After incubation for 48 h at 37 °C, the remaining cells in the upper chamber were removed and cells in the lower chamber were fixed with 4% polyformaldehyde and stained with 0.1% crystal violet. Images were taken with an optical microscope (Olympus, Tokyo, Japan).

### Fluorescence in situ hybridization (FISH)

The subcellular localization of lncRNA-Gm9866 was detected by FISH assays (Ribobio, Guangzhou, China). AML12 and JS-1 cells were fixed with 4% paraformaldehyde (Servicebio, Wuhan, China), prehybridized, and then immersed in hybridization solution containing the lncRNA-Gm9866 probe (Ribobio, Guangzhou, China) marked with cyanine 3 (Cy3) and Fam98b probe (GenePharma, Suzhou, China) marked with Fam98b, incubated them overnight at 37 °C. Then, cells were stained with 4′,6-diamidino-2-phenylindole (Ribobio, Guangzhou, China). Images were then acquired by laser scanning confocal microscopy (TCS SP8, LEICA, Germany).

### RNA immunoprecipitation (RIP) assay

RIP was performed using the RNA Immunoprecipitation Kit (Geneseed, Guangzhou, China) according to the manufacturer’s instructions. Briefly, 1 × 10^7^ AML12 cells were collected, centrifuged at 1000 rpm for 5 min, washed twice with cold PBS, and centrifuged again. The supernatant was discarded, buffer A (containing 1% volume protease inhibitor and 1% volume RNase inhibitor) was added and cells were lysed on ice for 10 min. Samples were centrifuged and 100 µL of the supernatant was used the Input control (tube 1). The remaining 900 µL was added to protein A + G beads, rotated 10 min at 4 ˚C and 10 revolutions/min. A magnetic separator was used to remove the supernatant, buffer A and buffer D were added and the samples were rotated 30 min at 4 ˚C and 10 revolutions/min. A magnetic separator was used to remove the supernatant, buffer A was added, and the sample was divided into two tubes. Again, a magnetic separator was used to remove the supernatant and buffer A, Fam98b antibody (Santa Cruz, California,USA, sc-398375), or IgG (5 µg each), was added and incubated 2 h at 4 ˚C, 10 revolutions/min. A magnetic separator was used to remove the supernatant and 350 µL buffer A and 450 µL of cell lysis supernatant was added to the magnetic beads and the reactions were rotated overnight at 4 ˚C and 10 rpm. After the reaction, a magnetic separator was used to collect the supernatant; DNA was removed and RNA was purified. Purified RNA was detected by qPCR.

### Cell transduction

Overexpression and knockdown of lncRNA-Gm9866 in AML12 and JS-1 cells was achieved by the transfection of lentiviruses (Genechem, Shanghai, China). The AML12 and JS-1 cells were seeded into a T25 culture flask separately, cultured overnight, and washed with PBS. Then, 2 mL of culture medium without penicillin/streptomycin was added to each bottle. Lentivirus was directly added to the culture medium (AML 12, MOI = 20; JS-1, MOI = 80). After 16 h, the medium containing lentivirus was discarded and replaced with new complete medium. After 72 h, puromycin was added to the medium for one week to screen stably infected AML12 and JS-1 cells. Finally, total RNA was extracted from stably infected AML12 and JS-1 cells to evaluate transfection efficiency. Silencing of Fam98b in AML12 cells was achieved by transfection of cells with a small interfering RNA (siRNA; Sangon Biotech, Shanghai, China) with Lipo6000 reagent (Beyotime Biotechnology, Shanghai, China) according to the manufacturer’s instructions.

### mRNA stability

AML12 cells were seeded in 6-well plates overnight, and treated with 5 μg/mL actinomycin D (ActD) (Beijing Noble Ryder Technology Co. Ltd., Beijing, China) to inhibit further RNA synthesis. AML12 cells were treated at different time intervals in the presence of actinomycin D (0, 3, 6, 9, 12 or 24 h). Total RNA was extracted and analyzed by qPCR. The remaining RNA levels of interest at each time point were normalized to that at the beginning (0 h).

### RNA extraction and quantitative polymerase chain reaction (qPCR)

Total RNA was isolated from JS-1 and AML12 cells by homogenizing liver tissues with a NucleoZOL isolation kit (Macherey–Nagel, Düren, Germany) in accordance with the manufacturer’s protocol. qPCR assays were performed using Prime ScriptTM RT Master Mix (Perfect Real Time) reagent kits (TaKaRa Bio, Shiga, Japan), along with a FastStart Universal SYBR Green Master (ROX) kit (Roche, Mannheim, Germany), according to the manufacturer’s instructions. The qPCR conditions were as follows: one cycle of 50 ℃ for 2 min, and 95 ℃ for 10 min, 40 cycles of 15 s at 95 ℃, and 1 min at 60 ℃. The expression of the target gene mRNA was normalized to that of GAPDH. The sequences of the primers used in this study were listed in Table 1. At least three independent experiments were carried out for each experimental condition.

**Table 1:** Primers sequences

Gene	Primer	Sequence
*lncRNA-Gm9866*	Forward	TGGTTGCTTGTTGATGCCTCCTG
	Reverse	CATCAGGACAAGCAGCGGTATCAG
*Fam98b*	Forward	TGTCAGGCACCCGTCCCATAAG
	Reverse	GGTGATCTGACGCTCTGAGG
*GAPDH*	Forward	GGTTGTCTCCTGCGACTTCA
	Reverse	TGGTCCAGGGTTTCTTTACTCC
*MMP9*	Forward	CAAAGACCTGAAAACCTCCAAC
	Reverse	GACTGCTTCTCTCCCATCATC
*TIMP1*	Forward	GCAAAGAGCTTTCTCAAAGACC
	Reverse	CTCCAGTTTGCAAGGGATAGAT
*BAX*	Forward	TTGCCCTCTTCTACTTTGCTAG
	Reverse	CCATGATGGTTCTGATCAGCTC
*BAD*	Forward	GAAGACGCTAGTGCTACAGATA
	Reverse	CTGCTGATGAATGTTGCTCC
*α-SMA*	Forward	CGTGGCTATTCCTTCGTGACTACTG
	Reverse	CGTCAGGCAGTTCGTAGCTCTTC
*COL-1*	Forward	GACAGGCGAACAAGGTGACAGAG
	Reverse	CAGGAGAACCAGGAGAACCAGGAG

### Western blot analysis

RIPA buffer (Solarbio, Shanghai, China) was used to lyse cells and a BCA kit (Beyotime, Shanghai, China) was used to quantify protein levels. The protein concentration was about 50 mg/mL, which was separated by 10% SDS-PAGE gel. β-actin (1:5000, AbMART, Shanghai, China, M2009) was used as a loading control. The antibody Fam98b (1:2000, FineTest, Wuhan, China, FNab03001), α-SMA (1:2000, Abcam, Cambridge, UK, ab124964), Collagen I (1:2000, Abcam, Cambridge, UK, sc-59772), Smad2/3 (1:2000, Abcam, Cambridge, UK, ab63672), BAD (1:2000, AbMART, Shanghai, China, 67830-1-Ig), BAX (1:2000, AbMART, Shanghai, China, T40051), Bcl2 (1:2000, AbMART, Shanghai, China, T40056), TGFβ (1:500, Wanleibio, Shenyang, China, WL02998), P-Smad2/3 (1:1000, Absin, Shanghai, China, abs131873), NOTCH3 (1:1000, Proteintech, Wuahn, China, 55114-1-AP) were used as primary antibody. The secondary antibodies were obtained from Invitrogen (1:20,000, Thermofisher, SA5-35521, SA5-35571). An Odyssey two-color infrared laser imaging system (LI-COR Biosciences, Lincoln, NE, USA) was employed to scan the blots. The quantitative analysis of grey values was performed using Image J software (NIH, USA).

### Statistical analysis

Data are present the mean ± standard deviation triplicate independent experiments. SPSS, version 25.0 (SPSS, Chicago, IL, USA) and GraphPad Prism (GraphPad software Inc.) was utilized for all statistical testing. Data were compared with the Student’s *t*-test and one-way analysis of variance (ANOVA). Probability (p)-values < 0.05 were considered statistically significant.

## Results

### LncRNA-Gm9866 expression in liver fibrosis in vivo and in vitro

**Fig. 1:**
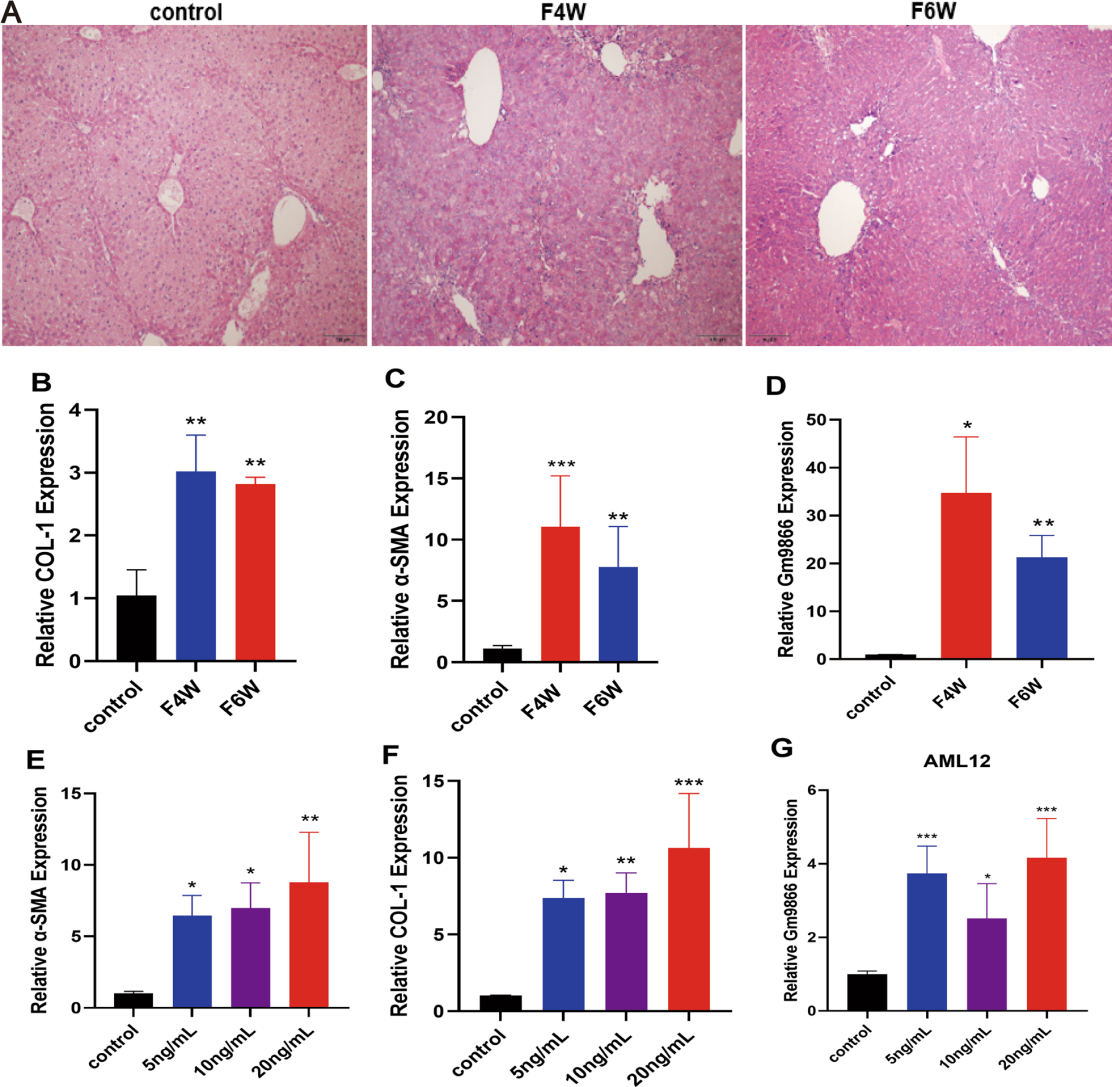
Expression level of lncRNA-Gm9866 in the induction of liver fibrosis. **A** Histological images of mouse livers stained with hematoxylin and eosin (scale bar: 50 μm). **B**, **C** Relative expression levels of COL-1 and α-SMA in liver tissue. **D** Expression levels of lncRNA-Gm9866 in different stages of fibrotic liver tissue. **E**, **F** Relative expression levels of α-SMA and COL-1 in AML12 hepatocytes. **G** LncRNA-Gm9866 expression level in AML12 hepatocytes stimulated with different concentrations of TGFβ1. F4W and F6W represented fibrosis modeling administration for 4 and 6 weeks. *P < 0.05, **P < 0.01, ***P < 0.001

To systematically verify that lncRNA-Gm9866 is related to liver fibrosis, we established CCl4-induced mouse liver fibrosis and in vitro cell models. Liver fibrosis was induced in C57BL/6 mice by injection of CCl4 for 4 and 6 weeks. As shown in Fig. 1A, hematoxylin and eosin (H&E) staining confirmed the presence of liver fibrosis. Compared with the control liver, α-SMA and COL-1 mRNA was significantly upregulated in the fibrotic liver (Fig. 1B, C). qPCR was performed to detect the expression level of lncRNA-Gm9866 in fibrotic and control livers; the level of lncRNA-Gm9866 in the fibrotic liver was upregulated (Fig. 1D). To construct a cellular fibrosis model, AML12 mouse hepatocytes were stimulated with different concentrations of TGFβ1, and mRNA levels of the pro-fibrogenic genes α-SMA and COL-1 were assessed. The relative expression levels of α-SMA and COL-1 in AML12 cells were significantly increased (Fig. 1E, F). Similarly, lncRNA-Gm9866 was upregulated in hepatocytes stimulated by TGFβ1(Fig. 1G).

### LncRNA-Gm9866 regulates expression of ECM genes in HSCs

**Fig. 2:**
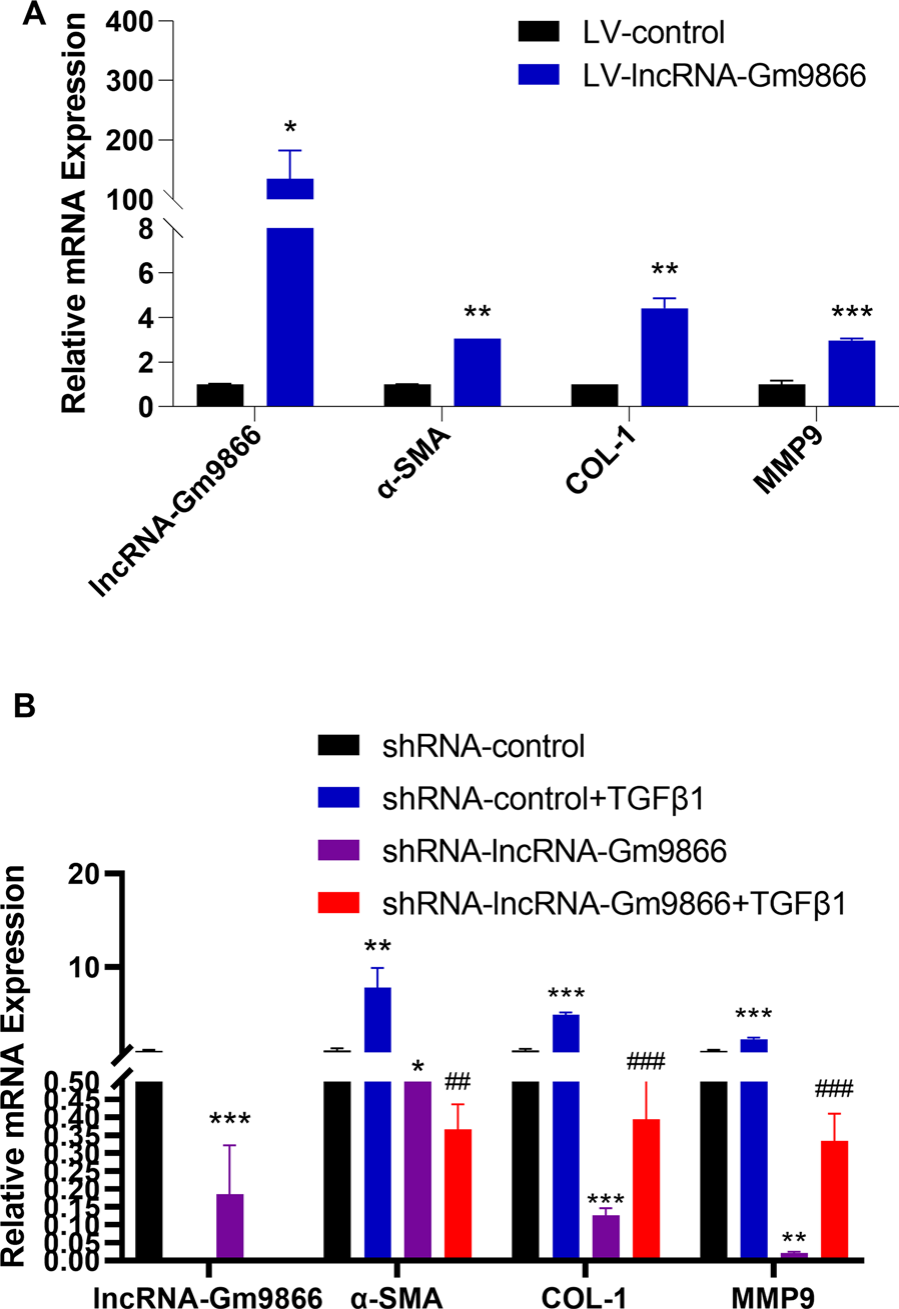
LncRNA-Gm9866 regulates the expression of extracellular matrix genes in HSCs. **A** mRNA levels of α-SMA, COL-1, and MMP9 in lncRNA-Gm9866-overexpressing JS-1 cells by qPCR. **B** JS-1 cells were infected with lentivirus-control or lentivirus-mediated shRNA-Gm9866 for 72 h and further treated with 10 ng/mL TGFβ1 for an additional 24 h. Expression of lncRNA-Gm9866, α-SMA, COL-1, and MMP9 was detected by qPCR. */^#^P < 0.05, **/^##^P < 0.01. *P < 0.05 vs. shRNA-control, *P < 0.05 vs. LV-control in **A** and ^#^P < 0.05 vs. shRNA-control + TGFβ1 in **B**

Activated HSCs are the primary producers of new matrix in fibrosis, characterized by the expression of α-SMA and the production of ECM are enhanced. In addition, activated HSCs can increase the expression of various fibrosis markers, including Col1α1. Col1α2. Col3α1. Col4α5, MMP2, MMP9, MMP10, TIMP1, and TGF β [1, 8]. To further evaluate the functional role of lncRNA-Gm9866 in liver fibrosis, lentiviruses overexpressing lncRNA-Gm9866 and knockdown of lncRNA-Gm9866 were transfected into the mouse hepatic stellate cell line JS-1. After overexpression of lncRNA-Gm9866, pro-fibrogenic genes such as α-SMA, COL-1, and MMP9 in JS-1 were significantly increased (Fig. 2A). In addition, we found that cells infected with lncRNA-Gm9866-shRNA expressed lower levels of α- SMA, COL-1 and MMP9. Moreover, knockdown of lncRNA-Gm9866 significantly decreased TGFβ1 induced upregulation of these pro-fibrogenic genes in HSCs (Fig. 2B). Taken together, these results indicate that lncRNA-Gm9866 promotes the expression of pro-fibrogenic genes and activation of HSCs.

### Knockdown of lncRNA-Gm9866 reduces TGFβ1-induced HCs apoptosis

**Fig. 3:**
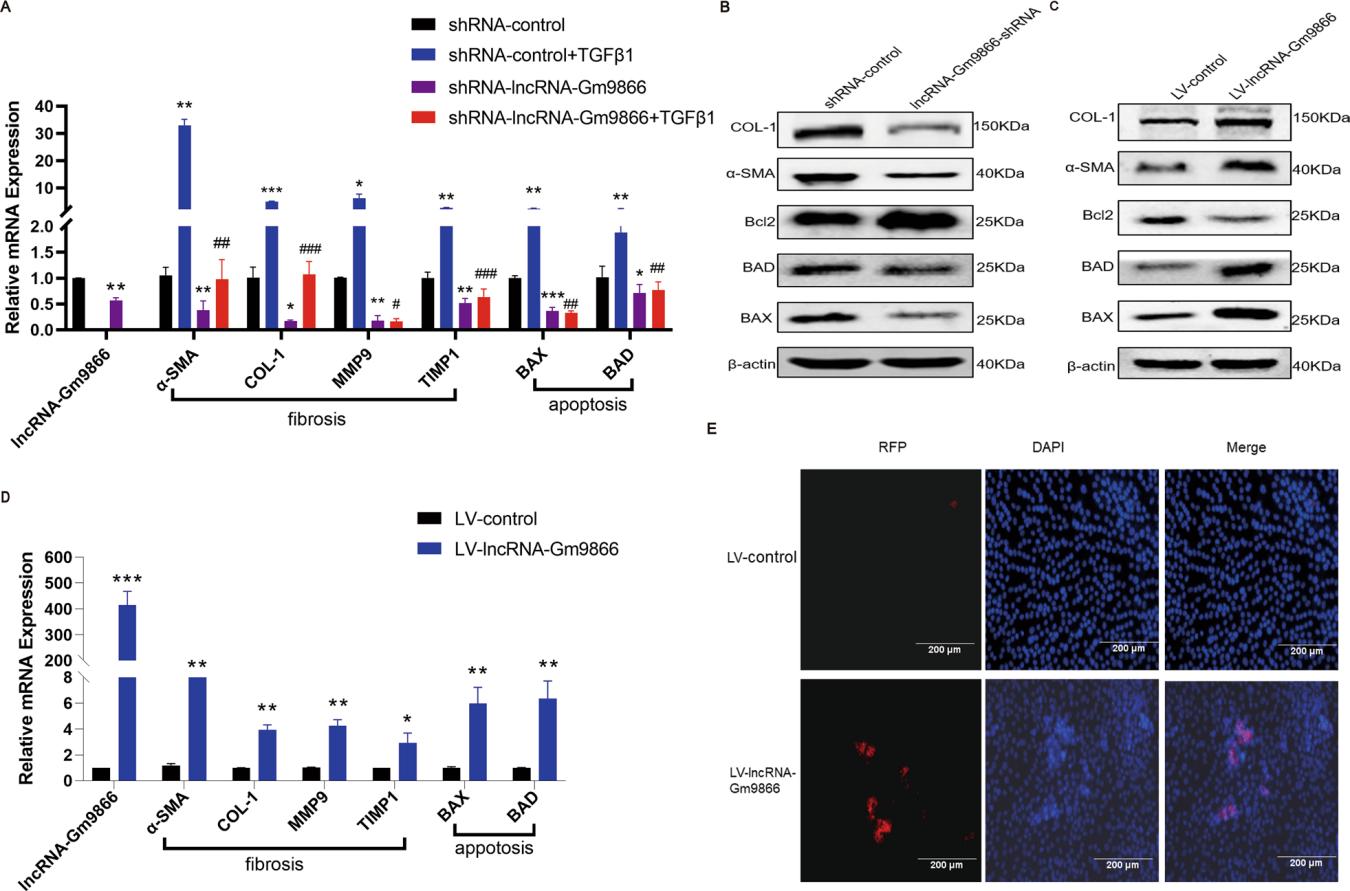
Knockdown of lncRNA-Gm9866 reduces TGFβ1-induced HCs apoptosis. **A** qPCR showed that knockdown of lncRNA-Gm9866 in AML12 cells abolished TGFβ1 (10 ng/mL, 24 h) induced upregulation of pro-fibrogenic genes α-SMA, COL-1, MMP9, and TIMP1 and apoptosis-related genes BAX and BAD. **B**, **C** Protein levels of α-SMA, COL-1, BAX, BAD, and Bcl2 were detected in lncRNA-Gm9866 knockdown and overexpressing AML12 cells by western blot. **D** mRNA levels of α-SMA, COL-1, MMP9, TIMP1, BAX, and BAD were detected in lncRNA-Gm9866-overexpressing AML12 cells by qPCR. **E** TUNEL assay indicated that overexpression of lncRNA-Gm9866 promoted apoptosis in AML12 cells (scale bar: 200 µm). */^#^P < 0.05, **/^##^P < 0.01, ***/^###^P < 0.001. *P < 0.05 vs. LV-control in **D** and *P < 0.05 vs. shRNA-control, ^#^P < 0.05 vs. shRNA-control + TGFβ1 in **A**

During liver injury, proinflammatory mediators, growth factors, and cytokines produced by cell injury and stimulation of immune cells activate mesenchymal precursor cells in tissues and induce their transdifferentiation into myofibroblasts. The phagocytosis of HSCs to apoptotic HCs or lymphocytes also directly triggers activation of fibrosis. Therefore, HCs apoptosis is an important initiating factor in the process of liver fibrosis [23]. Previous study found that overexpression of lncRNA-LFAR1 increased the expression of fibrosis markers in AML12 cells, while knocking down lncRNA-LFAR1 blunt TGFβ-induced dysregulation of apoptosis related genes such as Bax, BAD, and Bcl-XL in AML12 cells [8]. To investigate the role of lncRNA-Gm9866 in HCs, we first knocked down lncRNA-Gm9866 in AML12 cells and after confirming lncRNA-Gm9866 knockdown was effective, treated AML12 cells transfected with lncRNA-Gm9866-shRNA or shRNA control virus with TGFβ1. After TGFβ1 treatment, expression of pro-fibrogenic genes in HCs was upregulated, while knockdown of lncRNA-Gm9866 abolished TGFβ1-induced upregulation of these genes (Fig. 3A). Western blot further confirmed these findings and demonstrated that when AML12 cells were transfected with lncRNA-Gm9866 shRNA, expression of α-SMA and COL-1 were both decreased (Fig. 3B). On the other hand, overexpression of lncRNA-Gm9866 increased the expression of pro-fibrogenic genes in AML12 cells as assessed by qPCR and western blot (Fig. 3C).

We then investigated the effect of lncRNA-Gm9866 on HCs apoptosis. Overexpression of lncRNA-Gm9866 increased the expression of apoptosis-related genes in AML12 cells (Fig. 3D); these findings were further confirmed by western blot (Fig. 3C) and TUNEL analysis (Fig. 3E). TGFβ1 stimulation significantly increased AML12 apoptosis, and lncRNA-Gm9866 silencing significantly inhibited TGFβ1-induced AML12 apoptosis (Fig. 3A). Taken together, our results show that knockdown of lncRNA-Gm9866 decreased TGFβ1 induced apoptosis of HCs. Overexpression of lncRNA-Gm9866 promoted the expression of pro-fibrogenic and apoptosis-related genes.

### LncRNA-Gm9866 inhibits proliferation and migration of AML12 cells

**Fig. 4:**
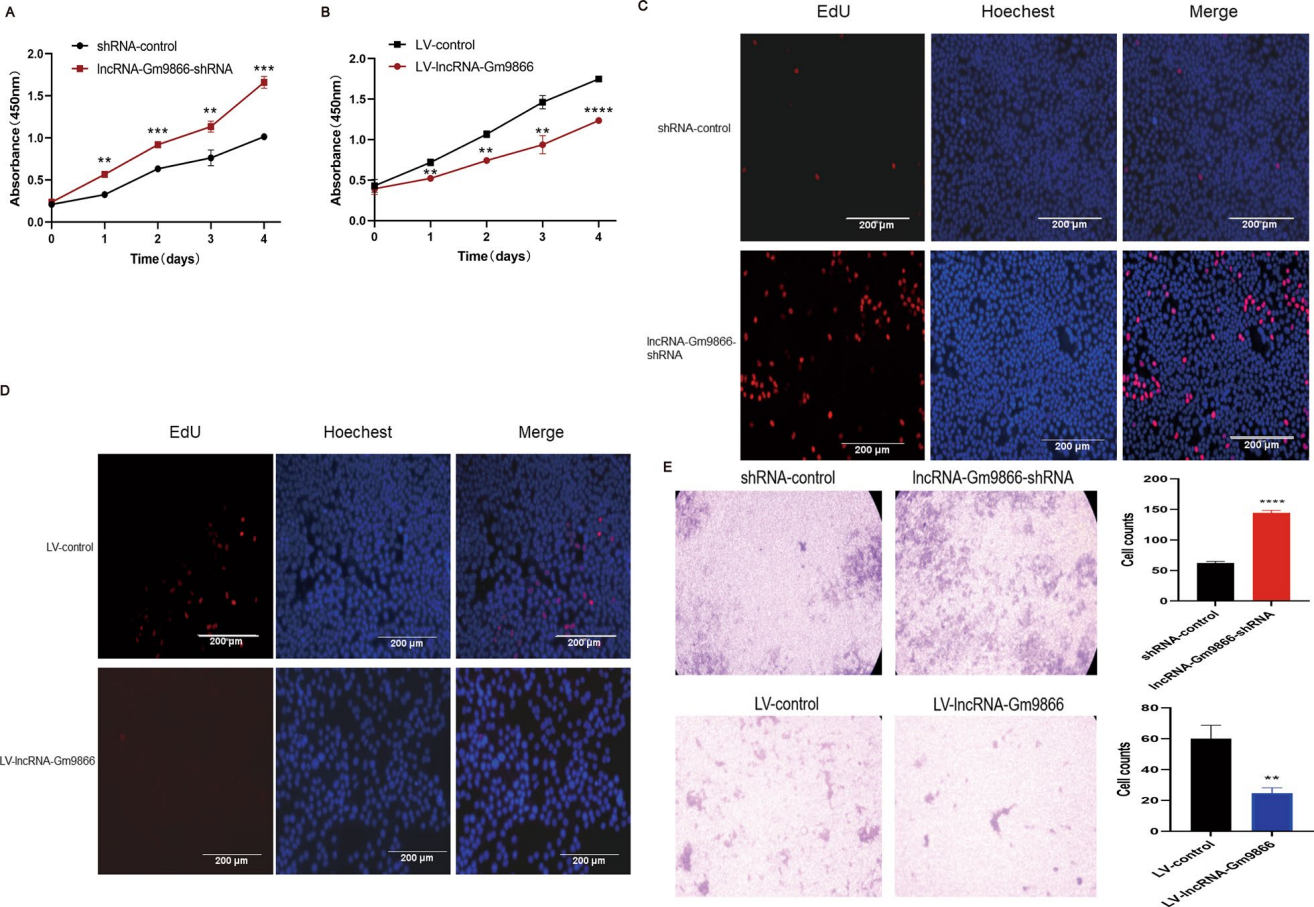
The effect of lncRNA-Gm9866 on the proliferation and migration of AML12 cells. **A**, **C** CCK8 and EdU assays show that silencing of lncRNA-Gm9866 promotes proliferation of AML12 cells. **B**, **D** CCK8 and EdU assays show that overexpression of lncRNA-Gm9866 inhibits proliferation of AML12 cells. **E** Migration assay shows that silencing of lncRNA-Gm9866 promotes the migration of AML12 cells while overexpression of lncRNA-Gm9866 inhibited AML12 cell migration. Scale bar, 200 µm. *P < 0.05, **P < 0.01, ***P < 0.001

To explore the effect of lncRNA-Gm9866 on the proliferation of AML12 cells, CCK-8 and EdU assays were performed. As shown in Fig. 4A–D, proliferation of AML12 cells was significantly reduced following lncRNA-Gm9866 overexpression and increased with silencing of lncRNA-Gm9866. Next, the effects of lncRNA-Gm9866 on the migration of AML12 cells was further explored. Similarly, the migratory ability of AML12 cells was inhibited following upregulation of lncRNA-Gm9866; migration was increased following downregulation of lncRNA-Gm9866 in AML12 cells (Fig. 4E). The liver has an amazing regenerative capacity to cope with hepatocyte death or loss of liver quality, and the increase in cell death may be a key driver of many chronic disease processes, including fibrosis and hepatocarcinogenesis [24]. Our results show that lncRNA-Gm9866 can inhibit proliferation and migration and promote apoptosis, confirming the pro-fibrotic effect of lncRNA-Gm9866.

### LncRNA-Gm9866 silencing inhibits CCl4-induced fibrosis

**Fig. 5:**
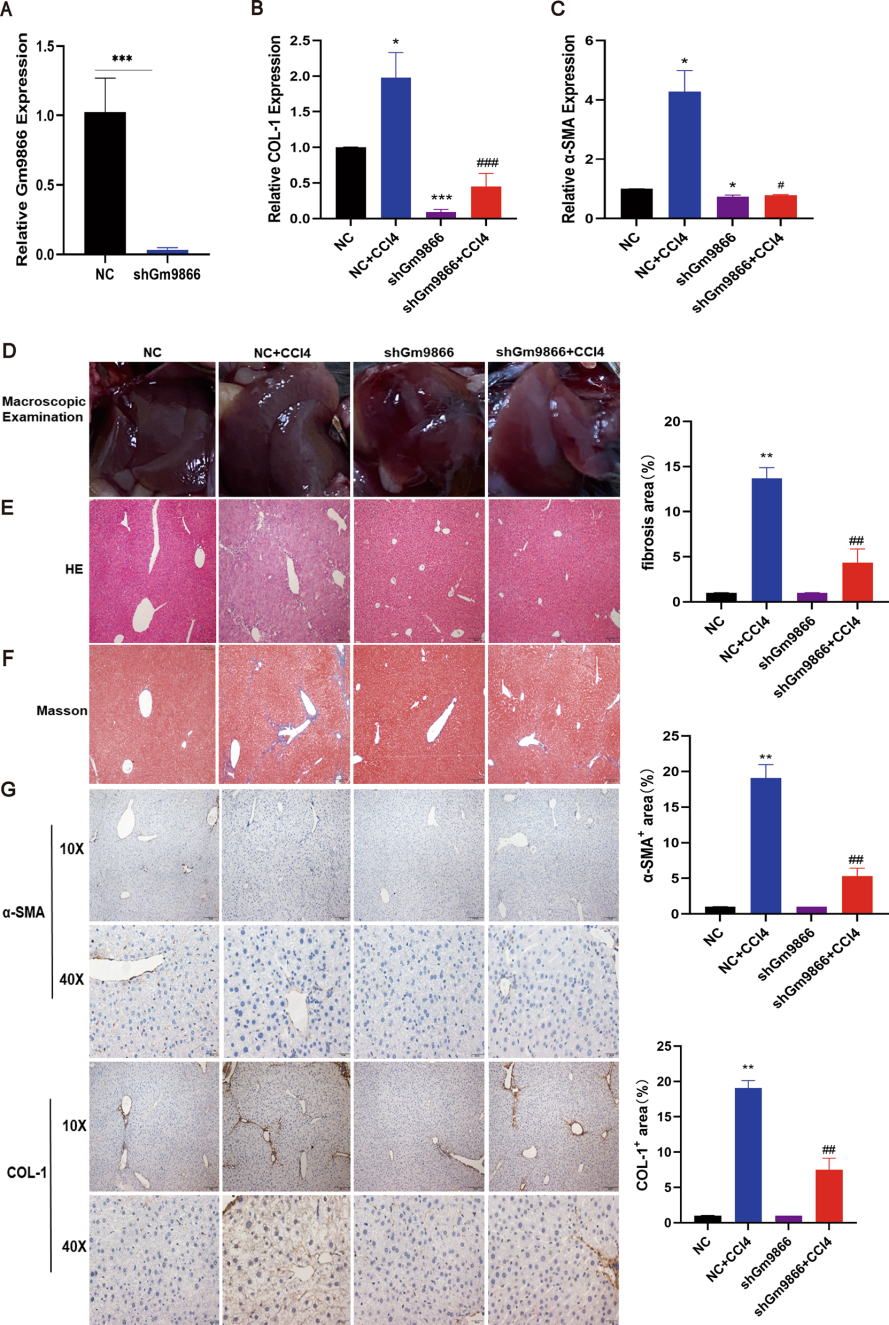
Knockdown of lncRNA-Gm9866 attenuates CCl4-induced liver fibrosis in vivo. **A** qPCR shows that lenti-shGm9866 interferes with expression of lncRNA-Gm9866 in vivo. **B** The relative expression level of COL-1 in liver tissue increased significantly in NC + CCl4 group and decreased significantly in shGm9866 + CCl4 group. **C** The relative expression level of α-SMA in liver tissue increased significantly in NC + CCl4 group and decreased significantly in shGm9866 + CCl4 group. **D** Macroscopic observation showed that the liver appearance of shGm9866 + CCl4 group was significantly smoother than that of NC + CCl4 group. **E** H&E staining showed that the morphology of hepatocytes and the structure of hepatic lobules in shGm9866 + CCl4 group were more regular and regular than those in NC + CCl4 group. **F** Masson staining showed that the deposition of shGm9866 + CCl4 collagen fibers was less than that of NC + CCl4. **G** IHC results showed that positive expression of α-SMA and COL-1 was significantly elevated in the NC + CCl4 group and decreased significantly in the shGm9866 + CCl4 group. ^*/#^ P < 0.05, ^**/##^P < 0.01, ^***/###^P < 0.001. *P stands for NC + CCl4 vs NC or shGm9866 vs NC, ^#^P stands for shGm9866 + CCl4 vs NC + CCl4

To explore the role of lncRNA-Gm9866 in liver fibrosis in vivo, lenti-shGm9866 or lenti-NC was injected into CCl4-treated mice through the tail vein one week after the first injection of CCl4. LncRNA-Gm9866 silencing was confirmed in whole liver extracts by qPCR (Fig. 5A). To further investigate whether the downregulation of lncRNA-Gm9866 in vivo alleviates liver fibrosis, we determined the degree of liver fibrosis in lentivirus-infected mice by various methods. The expression of fibrotic markers α- SMA and COL-1 in whole liver extracts were detected by qRT-PCR which shown that α-SMA and COL-1 in shGm9866 + CCl4 group were significantly lower than in NC + CCl4 group (Fig. 5B, C). Macroscopic observation of the appearance of mouse liver showed that the liver surface of NC + CCl4 group was significantly grainy, while the liver surface of shGm9866 + CCl4 group was smoother (Fig. 5D). H&E staining showed that the liver tissue structure of NC + CCl4 group mice was disordered, the structure of liver lobules was damaged, and pseudolobules were formed, while the liver tissue and liver lobule structure of shGm9866 + CCl4 group mice were normal (Fig. 5E). Masson staining showed that the collagen fibers in the liver tissue of NC + CCl4 group mice increased significantly, while only a small amount of collagen fibers were found in the liver tissue of shGm9866 + CCl4 group mice (Fig. 5F). Immunohistochemical staining results showed that α-SMA and COL-1 proteins were mainly expressed in the cytoplasm and stained brownish-yellow, and the number of positive expression cells of α-SMA and COL-1 proteins in the liver tissues of mice in the NC + CCl4 group increased significantly, while the liver tissues of mice in the shGm9866 + CCl4 group were less (Fig. 5G). To sum up, CCl4-treated lenti-NC infected mice developed severe liver fibrosis. However, as shown by macroscopic examination, H&E and Masson staining, IHC and qPCR for α-SMA and COL-1, administration of lenti-shGm9866 significantly mitigated CCl4-induced liver fibrosis. Taken together, our results indicate that lncRNA-Gm9866 knockdown can alleviate CCl4-induced liver fibrosis in vivo.

### LncRNA-Gm9866 affects liver fibrosis by interacting with Fam98b

**Fig. 6:**
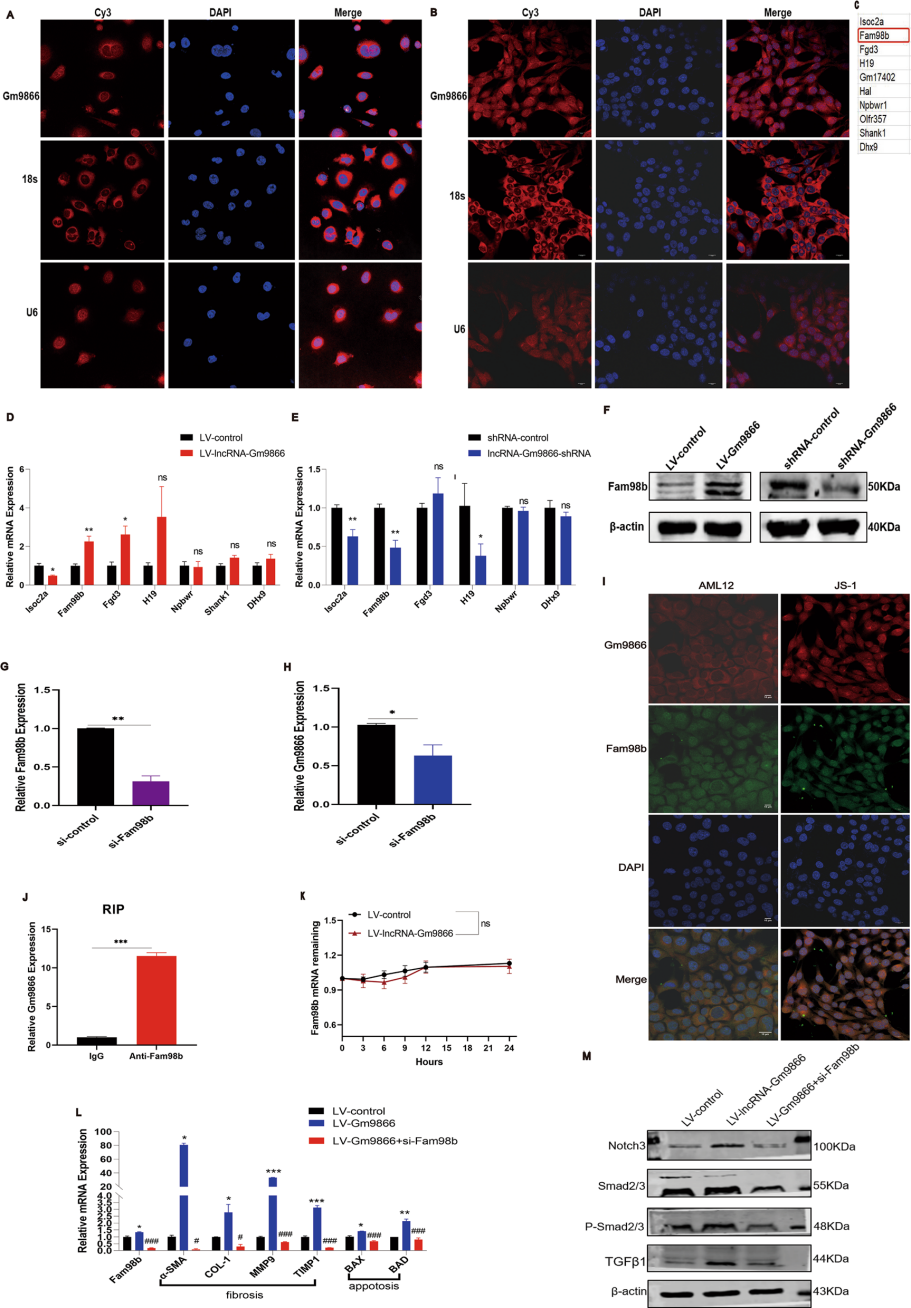
LncRNA-Gm9866 affects liver fibrosis by interacting with Fam98b. **A**, **B** FISH assay shows the subcellular localization of lncRNA-Gm9866 in AML12 and JS-1 cells (magnification, 630X). **C** Ten putative lncRNA-Gm9866 target genes identified with the lncRRIsearch database. **D**, **E** mRNA expression levels of 10 predicted genes after overexpression and silencing of lncRNA-Gm9866 in AML12 cells were detected by qPCR. **F** Protein level of Fam98b following overexpressing and silencing of lncRNA-Gm9866 in AML12 cells assessed by western blot. **G** qPCR confirmation of Fam98b interference in AML12 cells. **H** mRNA expression level of lncRNA-Gm9866 in AML12 cells following knockdown of Fam98b expression. **I** FISH assay shows lncRNA-Gm9866 is co-localized with Fam98b in AML12 and JS-1 cells (magnification, 630X). **J** Fold-enrichment of lncRNA-Gm9866 in RNAs of AML12 cells combined with Fam98b. **K** Actinomycin D experiment shows lncRNA-Gm9866 had no effect on the mRNA stability of Fam98b in AML12 cells. **L** mRNA level of pro-fibrogenic and pro-apoptosis genes after silencing of Fam98b in stably transfected AML12 cells compared with cells overexpressing lncRNA-Gm9866. **M** Protein level of pro-fibrogenic and pro-apoptosis genes after silencing of Fam98b in stably transfected AML12 cells compared with AML12 cells overexpressing lncRNA-Gm9866. */^#^P < 0.05, **/^##^P < 0.01, ***/^###^P < 0.001. *P < 0.05 vs. LV-control and ^#^P < 0.05 vs. LV-Gm9866 in **L**. ns, no significance

Long noncoding RNAs affect transcription and post-transcriptional regulation [14, 15], which have been linked to cellular localization. Therefore, we first confirmed the localization of lncRNA-Gm9866 in HCs and HSCs by FISH assay. Laser confocal microscopy showed that lncRNA-Gm9866 was expressed in the cytoplasm and nucleus of both cells, although mainly in the cytoplasm of AML12 cells (Fig. 6A, B), suggesting lncRNA-Gm9866 can exert biological functions via RNA interacting proteins. To investigate the exact mechanism by which lncRNA-Gm9866 regulates liver fibrosis, we next queried the lncRRIsearch database to identify 10 putative lncRNA-Gm9866 target genes (Fig. 6C). Expression levels of these 10 differentially expressed genes were verified using qPCR in AML12 cells overexpressing and silencing lncRNA-Gm9866 (Fig. 6D, E). Fam98b was significantly increased when lncRNA-Gm9866 was overexpressed, and decreased when lncRNA-Gm9866 was silenced. These findings were confirmed by western blot (Fig. 6F). Further, after knockdown of Fam98b in AML12 cells by RNA interference (Fig. 6G), we found that lncRNA-Gm9866 also decreased (Fig. 6H), suggesting an obvious interaction between lncRNA-Gm9866 and Fam98b. Interestingly, we found that lncRNA-Gm9866 co-localized with Fam98b in AML12 and JS-1 cells (Fig. 6I). Additionally, RIP analysis showed that lncRNA-Gm9866 could interact with Fam98b (P < 0.001, Fig. 6J). Since lncRNAs can regulate target molecule expression at the post-transcriptional level, we tested the effect of lncRNA-Gm9866 on Fam98b mRNA stability in AML12 cells. LncRNA-Gm9866 did not decrease the stability of Fam98b mRNA (P > 0.05, Fig. 6K). Taken together, these results indicate that lncRNA-Gm9866 directly interacts with Fam98b protein in liver fibrosis. However, as overexpression of lncRNA-Gm9866 does not affect the stability of Fam98b, we speculate that lncRNA-Gm9866 may regulate the transcription or maturation of Fam98b [25].

We then investigated the role of Fam98b in liver fibrosis. As mentioned above, overexpression of lncRNA-Gm9866 promotes the expression of pro-fibrogenic and pro-apoptosis genes, and lncRNA-Gm9866 can directly act on Fam98b. Whether silencing of Fam98b can reverse the pro-fibrogenic and pro-apoptosis effects of lncRNA-Gm9866 needs further study. Therefore, we conducted a rescue experiment in which Fam98b was silenced in stably transfected AML12 cells overexpressing lncRNA-Gm9866 to explore whether these conditions might alleviate fibrosis. qPCR results showed that after silencing Fam98b in the stably transfected AML12 cells, mRNA levels of the pro-fibrogenic genes α-SMA, COL-1, MMP9, and TIMP1 and the pro-apoptosis genes BAX and BAD were all decreased (Fig. 6L). These results were confirmed by western blot (Fig. 6M). Taken together, these results suggest silencing of Fam98b can rescue the pro-fibrogenic and pro-apoptosis effects of lncRNA-Gm9866, further indicating lncRNA-Gm9866 regulates liver fibrosis by targeting Fam98b.

### LncRNA-Gm9866/Fam98b modulates liver fibrosis by regulating activation of TGFβ/Smad and Notch pathways

**Fig. 7:**
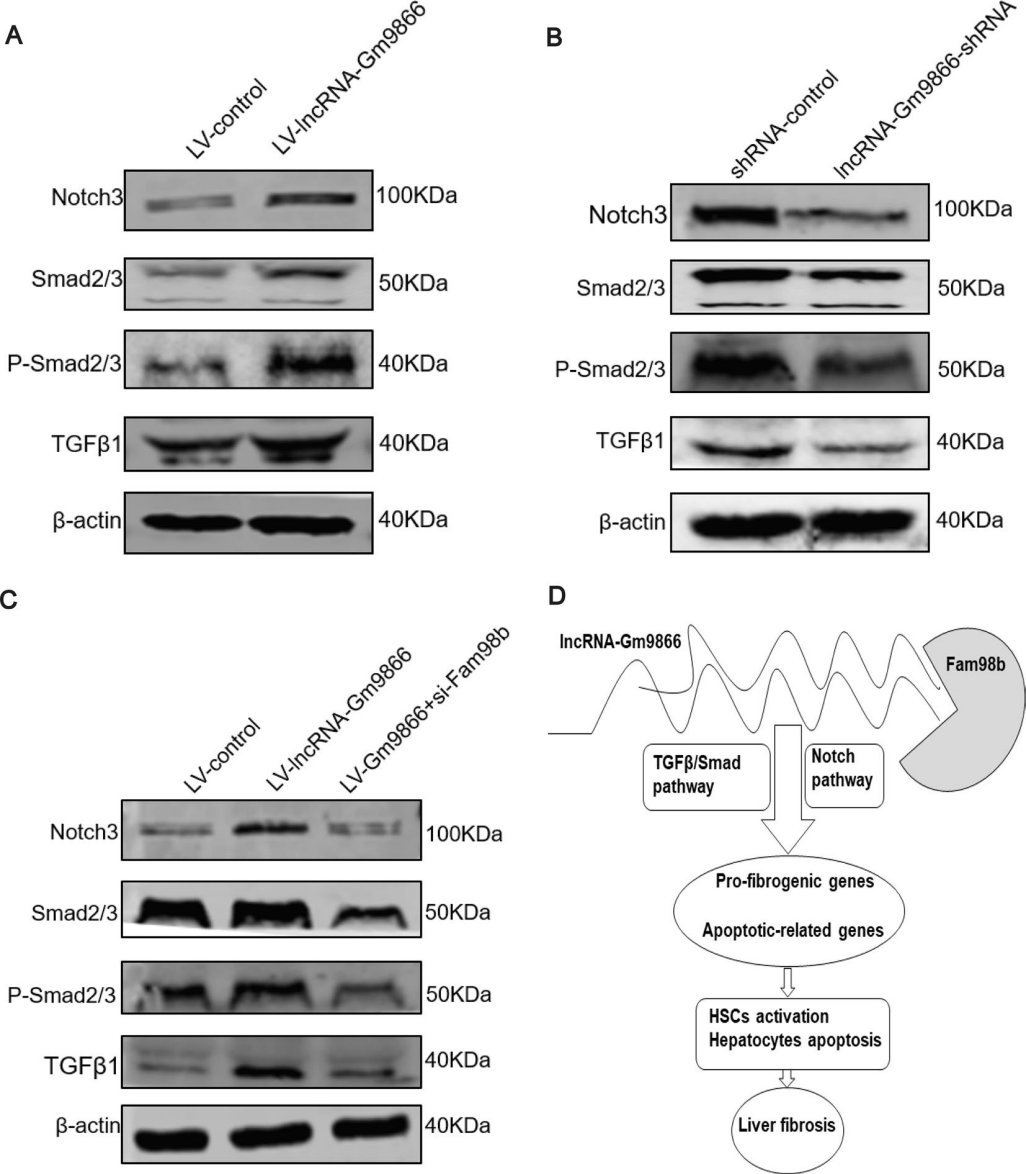
The lncRNA-Gm9866/Fam98b axis regulates activation of TGFβ/Smad and Notch pathways. **A**, **B** Effects of overexpressing and silencing lncRNA-Gm9866 in AML12 cells on the activation of TGFβ/Smad and Notch pathways. **C** Effects of Fam98b interference in stably transfected AML12 cells on inhibition of TGFβ/Smad and Notch pathways. **D** Schematic representation of the lncRNA-Gm9866/Fam98b/TGFβ/Smad/Notch pathways and their function in the progression of liver fibrosis

According to our results above, TGFβ affects the expression level of lncRNA-Gm9866, suggesting lncRAN-Gm9866 may regulate liver fibrosis via the TGFβ/Smad pathway. Therefore, we detected key pathway proteins in AML12 cells overexpressing or silencing lncRNA-Gm9866 and found that the expression levels of TGFβ1, Smad2/3, and P-Smad2/3 increased after overexpressing lncRNA-Gm9866 and decreased after silencing of lncRNA-Gm9866 (Fig. 7A, B). Liver fibrosis is a complex systemic pathological process involving multiple signaling pathways, including TGFβ/Smad, Hedgehog and Notch pathways [8]. To explore whether lncRNA-Gm9866 also regulates another pathway, we detected Notch3, a key protein in the Notch pathway, in AML12 cells with overexpression or silencing of lncRNA-Gm9866. We found that Notch3 increased when lncRNA-Gm9866 was overexpressed and decreased after silencing of lncRNA-Gm9866, indicating lncRNA-Gm9866 also regulates the Notch pathway (Fig. 7A, B). To conduct the rescue experiment, we silenced Fam98b in the stable transgenic line overexpressing lncRNA-Gm9866 AML12 cells and found that expression levels of TGFβ1, Smad2/3, P-Smad2/3, and Notch3 proteins were lower than those in AML12 cells overexpressing lncRNA-Gm9866 (Fig. 7C). Thus, the lncRNA-Gm9866/Fam98b axis may modulate live fibrosis by regulating activation of TGFβ/Smad and Notch pathways.

## Discussion

Liver fibrosis is considered a necessary stage in the progression of cirrhosis to hepatocellular carcinoma; fibrosis is a reversible stage [26, 27]. As the molecular basis of liver fibrosis has not been fully elucidated, the identification of therapeutic targets has been limited. In this study, we provide evidence for the functional role of lncRNA-Gm9866 in liver fibrosis. Our data showed that lncRNA-Gm9866 regulates liver fibrosis in vitro and in vivo. In vitro experiments confirmed that lncRNA-Gm9866 acts on both hepatocytes and hepatic stellate cells, promoting activation of HSCs and apoptosis of HCs. We also showed that lncRNA-Gm9866 bound Fam98b, activated TGFβ and Notch pathways, and promoted Smad2/3 phosphorylation. All these data support our conclusion that lncRNA-Gm9866 has multiple effects on HSC activation, HC apoptosis, and liver fibrosis, thus supporting it as a potential target of fibrosis.

An increasing number of studies on long noncoding RNAs related to the pathogenesis of liver fibrosis have recently been published. Overexpression of lncRNA- ANRIL has been shown to inhibit the activation of HSCs [28]. A previous study showed that lncRNA-NEAT1 sponged miR-139-5p and promoted HSC activation to regulate liver fibrosis by targeting the β-catenin/SOX9/TGFβ1 pathway [29]. Although HSCs play a key role in liver fibrosis, HCs are the dominant cells in the liver; HC apoptosis and proliferation are the core links of liver fibrosis. Therefore, it is believed that the solution to liver fibrosis is to inactivate HSCs and inhibit the apoptosis of HCs [8, 9, 23]. Recently, Song et al. conducted animal studies and found that TB001, a dual glucagon like peptide-1 receptor/glucagon hormone receptor (GLP-1R/GCGR) agonist, alleviated liver fibrosis by blocking HC apoptosis [30]. Zhou et al. found that treatment with human umbilical cord mesenchymal stem cells (HUC-MSCs) could protect HCs, inhibit HC apoptosis, and alleviate BDL-induced liver fibrosis in mice by upregulating the expression of miR-148-5p [31]. Zhang et al. determined the lncRNA expression profiles in livers of fibrotic and normal mice by lncRNA microarray and qPCR. By analyzing the expression of lncRNAs in various tissues in detail, a liver enriched lncRNA-LFAR1 (liver fibrosis-related lncRNA1) was identified. Subsequent cell experiments linked lncRNA-LFAR1 with HSCs and HCs, thus regulating liver fibrosis [8]. It is reported that lncRNA-HSER alleviated hepatic fibrosis by inhibiting hepatocyte apoptosis and epithelial-mesenchymal transition [32]. lncRNA-Gm9866 is located at A2 on chromosome 12, which has four exons and three transcripts. Hi-C chromosome conformation data from the mouse brain showed that the lncRNA-Gm9866 locus is spatially near that of Sox11 (SRY-related HMG-box gene 11), indicating there may be a regulatory relationship between these two loci in the nervous system [33]. Recently, experiments involving a mouse cardiac hypertrophy model showed that lncRNA-Gm9866 was highly expressed in hypertrophic myocardium. lncRNA-Gm9866 formed a module with six other lncRNAs, participated in the construction of a lncRNA gene network for a cardiac hypertrophy model, and was shown to mediate the occurrence of cardiac hypertrophy [34]. Another study found that lncRNA-Gm9866 was involved in development of the mouse dorsal root ganglion. Knockout of lncRNA-Gm9866 has been shown to reduce the expression levels of Sox11 and delay the recovery process in nerve cells [35]. These studies suggest that lncRNA-Gm9866 is involved in the development and injury of some cells. However, there have been no reports of the role of lncRNA-Gm9866 in liver fibrosis. In the present study, we found that lncRNA-Gm9866 can regulate HSCs and HCs to regulate liver fibrosis.

Fam98b is a member of family with sequence similarity 98 and is highly expressed in colorectal cancer. Knockdown of Fam98b reduces proliferation and colony formation of cancer cells [36]. Fam98b was reported as one of the components of tRNA (transfer RNA) splicing ligase complex [37, 38], the cytosolic HCLE-DDX1-HSPC117-Fam98b complex will enter the nucleus under active transcription conditions, where it will bind newly synthesized RNA to mediate nucleocytoplasmic transport, and may then regulate the translation of related RNAs [39, 40]. Two structural homologues, Fam98a and Fam98b, are required for expression of protein arginine methyl transferase 1 (PRMT1, considered a potential oncogenic protein) [36]. A recent study showed that the enhanced promoter activity driven by the main allele is reduced after knocking down Fam98b, revealing the importance of Fam98b in expression of the TGFβ1 gene in breast cancer [41]. Recently, lncRNA-Gm9866 has been suggested to be involved in inflammation and apoptosis in visual impairments [42]. Although Fam98b has been reported in several tumor-related studies, it has not yet been reported in liver fibrosis. In the present study, using bioinformatics analysis, cell transfection experiments, and RIP assays, Fam98b was found to be an interacting protein of lncRNA-Gm9866. Silencing Fam98b reversed the pro-fibrogenic and pro-apoptosis effects of lncRNA-Gm9866 overexpression, further substantiating the role of lncRNA-Gm9866 in regulating liver fibrosis by targeting Fam98b. Finally, silencing of Fam98b inhibited activation of Notch3 and TGFβ/Smad phosphorylation, two pathways implicated in liver fibrosis.

This study, however, failed to explore the effect of Fam98b overexpression on liver fibrosis due to the rich expression of Fam98b in hepatocytes and the effect of inhibiting Fam98b in HSCs remained unknown. In addition, this study also did not explore the specific binding sites between lncRNA-Gm9866 and Fam98b, how lncRNA-Gm9866 regulates TGFβ/Smad and Notch pathways, and the relationship between Fam98b and TGFβ/Smad and Notch pathways. These should be further studied in future research through luciferase analysis and chromatin immunoprecipitation experiments.

## Conclusions

lncRNA-Gm9866 promotes liver fibrosis by interacting with Fam98b and regulating activation of TGFβ/Smad and Notch pathways. These results broaden the regulatory network of lncRNA-Gm9866, deepen our understanding of the molecular mechanism of liver fibrosis, and show that activation of lncRNA-Gm9866 regulation in HSCs and HCs may represent a novel target for controlling liver fibrosis.

## Data Availability

The data used to support the findings of this study are included within the article.

## Abbreviations

CCl4: Carbon tetrachloride
lncRNAs: Long noncoding RNAs
NC: Negative control
ActD: Actinomycin D
tRNA: Transfer RNA
ECM: Extracellular matrix
HSCs: Hepatic stellate cells
HCs: Hepatocytes
qPCR, qRT-PCR: Quantitative polymerase chain reaction
WB: Western Blot
TGFβ1: Transforming growth factor-β1
FISH: Fluorescence in situ hybridization
RIP: RNA binding protein immunoprecipitation
α- SMA: α-Smooth muscle actin
COL-1: Collagen I
IGFBPrP1: Insulin growth factor binding protein
ZEB1: E-box-binding homeobox 1
EpCAM: Epithelial cell adhesion molecule
SRY: Sex determining region Y
BAX: BCL2-associated X
BAD: Bcl-xL/Bcl-2asociated death promoter
MMP9: Matrix metalloprotein9
TIMP1: Tissue inhibitors of metalloproteinase 1
Bcl2: B-cell lymphoma-2
GLP-1R/GCGR: Glucagon like peptide-1 receptor/glucagon hormone receptor
HUC-MSCs: Human umbilical cord mesenchymal stem cells
PRMT1: Protein arginine methyl transferase 1
